# Classification of pulmonary diseases from chest radiographs using deep transfer learning

**DOI:** 10.1371/journal.pone.0316929

**Published:** 2025-03-17

**Authors:** Muneeba Shamas, Huma Tauseef, Ashfaq Ahmad, Ali Raza, Yazeed Yasin Ghadi, Orken Mamyrbayev, Kymbat Momynzhanova, Tahani Jaser Alahmadi

**Affiliations:** 1 Department of Computer Science, Lahore College for Women University, Lahore, Pakistan; 2 Department of Computer Science, MY University, Islamabad, Pakistan; 3 Department of Computer Science, Al Ain University, Abu Dhabi, United Arab Emirates; 4 Institute of Information and Computational Technologies, Almaty, Kazakhstan; 5 Department of Information Systems, College of Computer and Information Sciences, Princess Nourah Bint Abdulrahman University, Riyadh, Saudi Arabia; Najran University College of Computer Science and Information Systems, SAUDI ARABIA

## Abstract

Pulmonary diseases are the leading causes of disabilities and deaths worldwide. Early diagnosis of pulmonary diseases can reduce the fatality rate. Chest radiographs are commonly used to diagnose pulmonary diseases. In clinical practice, diagnosing pulmonary diseases using chest radiographs is challenging due to Overlapping and complex anatomical Structures, variability in radiographs, and their quality. The availability of a medical specialist with extensive professional experience is profoundly required. With the use of Convolutional Neural Networks in the medical field, diagnosis can be improved by automatically detecting and classifying these diseases. This paper has explored the effectiveness of Convolutional Neural Networks and transfer learning to improve the predictive outcomes of fifteen different pulmonary diseases using chest radiographs. Our proposed deep transfer learning-based computational model achieved promising results as compared to existing state-of-the-art methods. Our model reported an overall specificity of 97.92%, a sensitivity of 97.30%, a precision of 97.94%, and an Area under the Curve of 97.61%. It has been observed that the promising results of our proposed model will be valuable tool for practitioners in decision-making and efficiently diagnosing various pulmonary diseases.

## 1. Introduction

Pulmonary diseases like Tuberculosis, Chronic Obstructive Pulmonary Disease (COPD), Asthma, Pneumonia, Lung Nodules, etc. are among the leading causes of disabilities and deaths worldwide. Around 10 million people suffer from Tuberculosis each year, causing a death toll of 1.5 million annually [1]. COPD is the third most common disease that causes death, affecting 65 million people and causing around 3 million deaths per year [2]. Almost 262 million people are infected by Asthma, resulting in about 461,000 deaths yearly [3]. Early detection of lung diseases can reduce their fatality rate. To diagnose pulmonary diseases, Computed Tomography (CT) [4], Magnetic Resonance Imaging (MRI) [5], Ultrasounds [6], Nuclear Lung Imaging [7], Positron Emission Tomography (PET) [8], and radiographs (X-Rays) [9] are used.

Among all these methods, radiographs are most commonly used for diagnosis because they can reveal some unknown changes happening in the human body due to diseases, are cost-effective, have low radiation dosage, and are non-invasive. Due to the common use of chest radiographs, a vast number of publicly available chest X-ray datasets are present. Some of these datasets include the Japanese Society of Radiological Technology (JSRT) dataset [10], the Open-i Indiana University Chest X-Ray dataset [11], the Shenzhen Hospital X-ray dataset [12], the Montgomery County X-ray dataset [12] and the National Institutes of Health ChestX-ray8 dataset [13].

In clinical practice, it is a challenging task to diagnose pulmonary diseases using chest X-rays. It heavily depends on the availability of a medical specialist with years of professional experience. Hence, Computer Aided Detection (CAD) systems are needed that automatically detect and classify pulmonary diseases by simply reading chest radiographs. In addition, they help medical specialists to make quantitative decisions. This is done by transferring man’s knowledge to machine intelligence.

Previously machine learning-based CAD systems were used for detecting and classifying pulmonary diseases using chest radiographs due to the non-availability of large enough datasets. However, with the release of the National Institutes of Health ChestX-ray8 dataset, compromising 108,948 frontal-view chest radiographs, work is now being done by applying deep learning techniques to CAD systems. These CAD systems are used for detecting and classifying pulmonary diseases using chest radiographs with higher precision. Recently transfer learning is also becoming a very popular approach to developing CAD systems. It involves using the knowledge acquired for solving one problem, to solve another problem. One of the benefits of transfer learning is that it requires comparatively less data. For example, in transfer learning, Convolutional Neural Networks (CNNs) pre-trained on the ImageNet Dataset [14] as part of the ImageNet Large Scale Visual Recognition Challenge (ILSVRC) [15] are used for the classification of other datasets. Transfer learning has given good accuracy in detecting and classifying pulmonary diseases using chest radiographs, but there is still much room for improvement.

This paper aims to propose an improved model for the detection and classification of pulmonary diseases using a radiographic dataset. An end-to-end structural model has been proposed in this paper, that has very little execution time and does not use manual selection and feature extraction methods. So, the main contributions of this work are summarized as follows:

- This work explores the use of Convolutional Neural Networks (CNNs) and transfer learning in the medical field, specifically for diagnosing pulmonary diseases from chest radiographs. By leveraging the power of deep learning algorithms and adding fully connected layers tailored to classify different classes of pulmonary diseases with enhanced accuracy.
- This research introduces a novel deep transfer learning-based model designed to detect and classify pulmonary diseases from chest radiographs. This study utilizes a feature extraction technique on a pre-trained Visual Geometry Group 16 (VGG16) model and adds new fully connected layers tailored specifically to classify pulmonary diseases from chest radiographs.
- The proposed model achieves highly promising results in terms of key evaluation metrics. With an overall specificity of 97.92%, a sensitivity of 97.30%, a precision of 97.94%, and an Area under the Curve (AUC) of 97.61%, the model demonstrates a high level of accuracy in detecting and classifying pulmonary diseases from chest radiographs.
- The generalized approach of our proposed model can classify all fifteen different pulmonary diseases, with improved performance than existing state-of-the-art models.

The structure of the remaining paper is as follows: Section 2 covers the detailed literature review of existing methods and models adopted to perform the detection and classification of pulmonary diseases. Section 3 elaborates on the datasets used in this paper. It also discusses the preprocessing steps and the architectural details of the proposed model in detail. Section 4 gives an analysis of the performance results of the proposed model. Results are also compared with other state-of-the-art models. Finally, section 5 concludes the research based on the analysis of results, and a discussion about future work is also included.

## 2. Literature review

Traditional methods require hand-crafted features for training a classifier [16]. It becomes very challenging to select the appropriate features and extract them robustly [17,18]. To overcome this problem, deep learning is now commonly used. It removes the challenge by automatically selecting and extracting the required features. It is smart enough to learn and make decisions on its own. In the field of pulmonary disease classification and detection, deep learning-based approaches and techniques are very common.

Numerous studies can be found in the literature that have used deep learning techniques for classifying pulmonary diseases. The results are very promising. A model called CheXNet was developed by Rajpurkar et al. [19]. It was a 121-layer Dense Convolutional Network (DenseNet) that had been initialized with weights of a model trained on ImageNet. CheXNet outperformed radiologists in detecting and classifying fourteen different pulmonary diseases from chest radiographs. Rubin et al. [20] developed a novel architecture called DualNet for the classification of pulmonary diseases using frontal and lateral radiographs. It consisted of two CNNs working in parallel for frontal radiographs and lateral radiographs, respectively. Hwang et al. [21] developed a deep CNN consisting of 27 layers with 12 residual connections to detect Active Pulmonary Tuberculosis. The model performed lesion-wise localization along with image-wise classification. The last layer of the model was split for this purpose.

Moreover, multiple classifiers can also be used at the same time to perform a combined task [22,23]. A CNN model was developed by Hwang et al. [24] for classifying and localizing Pneumothorax, Pneumonia, Active Tuberculosis, and Pulmonary Malignant Neoplasm using radiographs. They developed a model with dense blocks comprising five classifiers that worked in parallel. Four of them were dedicated to each disease, and the fifth one did the classification. Ozturk et al. [25] used deep CNN to detect COVID-19 using radiographs. They developed a You Only Look Once (YOLO) system, which detected real-time objects using a DarkNet model as a classifier. Khan et al. [26] designed a CoroNet model based on Xception architecture. This model identified COVID-19-infected radiographs. The model had 71 layers and was pre-trained on the ImageNet dataset and then trained end-to-end on the dataset that was used for this study. Instead of classical convolution layers, depth-wise separable convolution layers were used. Table 1 details some studies in which deep learning-based methods are used for detecting and classifying pulmonary diseases from chest radiographs.

**Table 1:** Existing studies on deep learning-based methods.

Paper	Dataset	Diseases	Preprocessing	Model
[27]	ChestXray14	12	Downscaled the images to 32 × 32	Custom Sequential CNN
[28]	ChestXray14	13	Resized the images to 128 × 128 × 3, normalized and augmented	DenseNet121, InceptionResNetV2, and ResNet152V2
[29]	Peru	4	–	GoogLeNet pre-trained on ImageNet
[30]	ChestXray14	14	–	ResNet18 pre-trained on ImageNet
[31]	Kermany	1	Resized the images to 224 × 224 × 3	VGG16
[32]	ChestXray14	1	Downscaled the images to 512 × 512	ResNet50 pre-trained on COCO
[33]	MIMIC-CXR	4	Downscaled the images to 512 × 512, cropped the lung ROIs	DenseNet pre-trained on ImageNet
[34]	Self-collected	1	Contrast-limited adaptive histogram equalization	YOLO Darknet19 pre-trained model
[35]	JSRT Dataset	1	Downscaled the images to 256 × 256, lung segmentation using UNet-based CNN	Custom Sequential CNN
[36]	UCSF	14	Downscaled the images to 256 × 256	GoogLeNet pre-trained on colored ImageNet
[37]	Stanford Radiology Dataset	14	Downscaled the images to 224 × 224, histogram equalization	GoogLeNet
[38]	Montgomery County Set	1	Downscaled the images to 512 × 512	Feed-forward neural network with backpropagation tuning
[39]	Joseph Paul Cohen, and ChexPert dataset	1	–	ResNet18, ResNet50, SqueezeNet, and DenseNet-121 pre-trained on ImageNet

There are a few challenges faced while detecting and classifying pulmonary diseases using chest radiographs. Available medical data is usually very limited and small in size. This is because clinical data is sometimes not permitted to be shared due to patient privacy laws. So it becomes a hurdle for researchers working on the analysis of medical data as a lot of data is often needed by deep learning models. This is being overcome by the use of transfer learning as pre-trained CNN models require smaller datasets for training [29,30,32,33]. Another challenge is that most networks trained are not general. They are specific for some diseases. However, some studies have successfully trained networks that can detect and classify multiple diseases [27,28,30]. Moreover, transfer learning has given good accuracy in detecting and classifying pulmonary diseases using chest radiographs, but there is still much room for improvement.

## 3. Materials and methods

### 3.1. Dataset description

The dataset for this paper was formed by selecting 6507 radiographs from three publicly available datasets: the Shenzhen Hospital X-ray Dataset, the Montgomery County X-ray Dataset, and the National Institutes of Health ChestX-ray8 Dataset. All the radiographs selected for this paper were of anteroposterior (AP) and posteroanterior (PA) views. Table 2 gives the details of the number of images selected for each class.

**Table 2:** Number of images selected for each class.

Class	Number of Images
Atelectasis	460
Cardiomegaly	414
Consolidation	406
Edema	407
Effusion	493
Emphysema	400
Fibrosis	401
Healthy Persons	406
Hernia	226
Infiltration	488
Mass	405
Nodule	402
Pleural Thickening	402
Pneumonia	400
Pneumothorax	403
Tuberculosis	394
**Total**	**6507**

**Fig 1:**
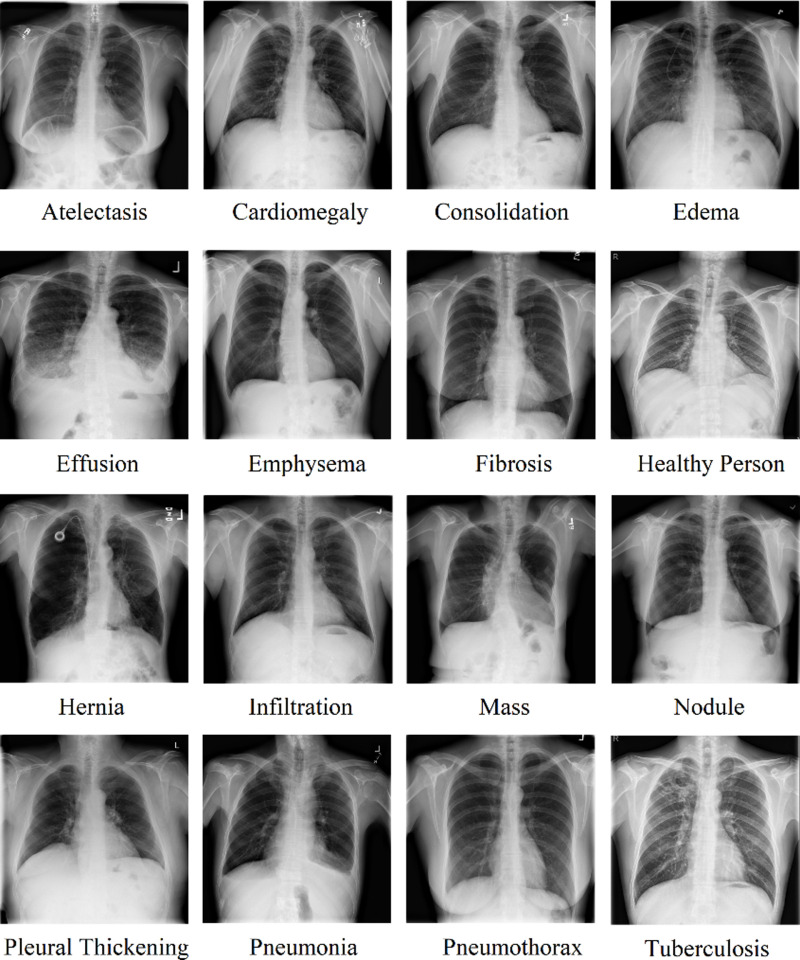
Representative chest radiographs of all classes.

Fig 1 shows representative chest radiographs of all classes. The dataset was randomly split into three independent datasets using the recommended [40] 70%, 15%, and 15% ratios for training, validation, and testing, respectively.

### 3.2. Methodology of the proposed model

**Fig 2:**
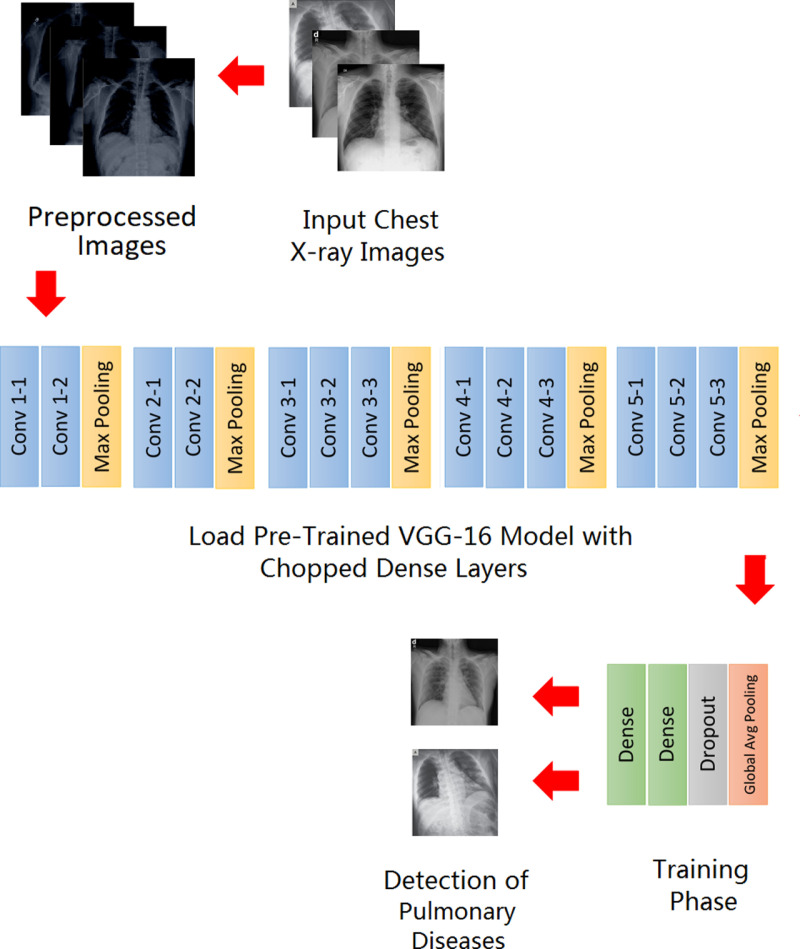
Schematic representation of the proposed model.

This section discusses the model’s architecture for detecting and classifying pulmonary diseases from chest radiographs. The proposed model uses the convolutional layers from the VGG16 [41] model for transfer learning, and a new combination of four fully connected layers has been added at the end as shown in Fig 2. In transfer learning, the model is first trained on a large, non-medical dataset, and the information learned from this model is transferred to the model to be trained on the medical dataset. Different ways to do transfer learning include fine-tuning and feature extraction. This paper uses the feature extraction technique.

**Fig 3:**
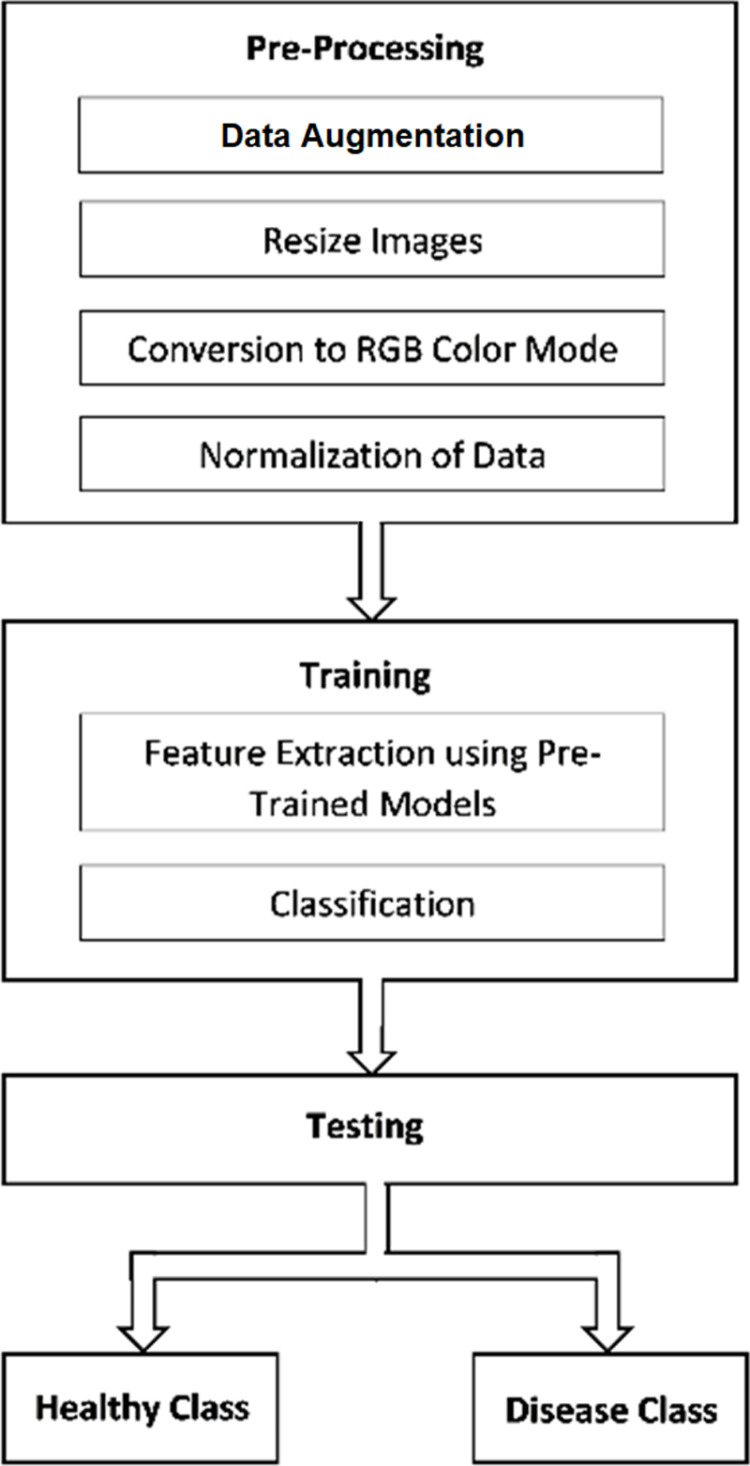
Flow of methodology.

Moreover, multiclass classification has been done through binary relevance, which is a technique that converts multiclass classification into binary classification. The dataset was divided into smaller datasets (one for each class), and then the model was trained for each class independently. Each smaller dataset consisted of two classes; the first was of each disease one by one, and the second was of healthy persons. The architecture of the proposed model has been divided into three different stages: preprocessing, feature extraction, and classification. Fig 3 shows the flow of the proposed methodology.

### 3.3. Preprocessing

There were only 226 images of Hernia found in the datasets, so data augmentation was done for this class. The images were augmented using three data augmentation methods; rotation, translation, and horizontal flip. 174 augmented images were randomly selected and added to the dataset. This made a total of 400 images of this class.

**Fig 4:**
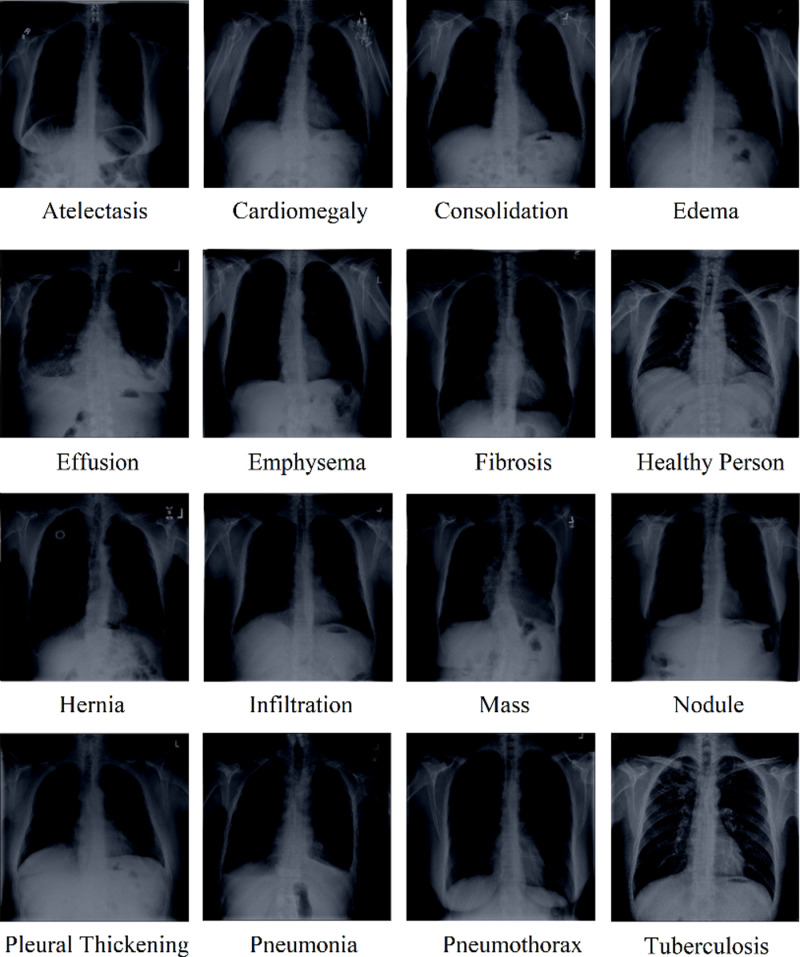
Representative chest radiographs of all classes after preprocessing.

Moreover, data was preprocessed to convert it to a standard format before passing it to the model. The size of images from different datasets was not the same, so all images were resized to 224 x 224 pixels. This small size was selected to speed up the processing and reduce heavy computation. Moreover, some of the images were originally in RGB color mode, while the others were in grayscale. So, all of them were converted to RGB color mode. Next, the mean RGB value of the ImageNet dataset [123.68, 116.78, 103.94] was subtracted from every pixel to normalize the data. Fig 4 shows representative chest radiographs of all classes after preprocessing. These preprocessed images were then used throughout the remaining study.

### 3.4. Feature extraction

The proposed model uses the VGG16 model for extracting features. This section describes the VGG16 model and its role in extracting features. The performance of VGG16 was also compared to pre-trained DenseNet121.

#### 3.4.1. Architecture of VGG16.

Karen Simonyan and Andrew Zisserman proposed a CNN model called VGG16 in 2014. Related background information is retained in the last convolutional layers of many other proposed CNN models, including AlexNet [42], which creates a disturbance in prediction. But VGG16 does not retain it, which helps it to get rid of this problem and make better predictions.

The architecture of VGG16 consists of five convolutional blocks having convolution and max pooling layers. An input of 224 × 224 × 3 is given as input to the model. It goes through the first two convolutional layers with filters of size 3 × 3 and 64 feature maps. This is the smallest possible filter size that can capture the notion of center, up/down, left/right. First, the dimensions change to 222 × 224 × 64. Then the maximum pooling layer reduced its dimension to 112 × 112 × 64 with a stride of 2 and a window of size 2 × 2 pixels. The same process is repeated four more times, reducing the dimensions to 7 × 7 × 512. For this paper, the layers till this point were initialized with weights from a model pre-trained on a large-scale publicly available ImageNet dataset, and the weights were frozen. The details parameter setting of the function of each layer of VGG16 is given in Table 3.

**Table 3:** Layer wise configuration details of Vgg16 model.

Layer (Type)	Output shape	Strides	Kernel	Pool size	Parameters	Activation function
Input Layer	224 × 224 × 3	–	–	–	0	–
Conv 2D	224 × 224 × 64	1	3 × 3	–	1792	ReLU
Conv 2D	224 × 224 × 64	1	3 × 3	–	36928	ReLU
Max Pooling 2D	112 × 112 × 64	2	–	2 × 2	0	–
Conv 2D	112 × 112 × 128	1	3 × 3	–	73856	ReLU
Conv 2D	112 × 112 × 128	1	3 × 3	–	147584	ReLU
Max Pooling 2D	56 × 56 × 128	2	–	2 × 2	0	–
Conv 2D	56 × 56 × 256	1	3 × 3	–	295168	ReLU
Conv 2D	56 × 56 × 256	1	3 × 3	–	590080	ReLU
Conv 2D	56 × 56 × 256	1	3 × 3	–	590080	ReLU
Max Pooling 2D	28 × 28 × 256	2	–	2 × 2	0	–
Conv 2D	28 × 28 × 512	1	3 × 3	–	1180160	ReLU
Conv 2D	28 × 28 × 512	1	3 × 3	–	2359808	ReLU
Conv 2D	28 × 28 × 512	1	3 × 3	–	2359808	ReLU
Max Pooling 2D	14 × 14 × 512	2	–	2 × 2	0	–
Conv 2D	14 × 14 × 512	1	3 × 3	–	2359808	ReLU
Conv 2D	14 × 14 × 512	1	3 × 3	–	2359808	ReLU
Conv 2D	14 × 14 × 512	1	3 × 3	–	2359808	ReLU
Max Pooling 2D	7 × 7 × 512	2	–	2 × 2	0	–

#### 3.4.2. Extraction of features.

VGG16 model was pre-trained on the ImageNet dataset. First, the weights of the original layers were frozen, and features were extracted from them. Only the weights of the newly added layers, that were used in the classification stage, were changed in the training process. The dimension of the final feature representation was 7 × 7 × 512, which was then given as input to the classifier.

### 3.5. Classification

A model consisting of a novel combination of four fully connected layers was designed and used for performing classification. This combination was selected after repeated experimentation. Features extracted from the VGG16 model were passed to this model, which did the classification. Train_test_split and validation_split were used to split the dataset into three parts; training, testing, and validation. The model had four layers, including a global average pooling layer, a dropout layer, and two dense layers. Global average pooling returns the average from the entire input for that layer, treating it as a single block. Since VGG16 is a very large network and is prone to over-fitting with less data, a dropout layer with a 0.2 rate was included. The dropout layer drops some neurons in each training cycle to avoid too much learning from training data. After dropout, a dense layer with 100 neurons and a ReLU activation function was added. The classification was binary, so the sigmoid activation function was used in the last dense layer. Table 4 shows the output shape, number of parameters, and activation function of each classifier layer.

**Table 4:** Layer wise configuration details of proposed classifier.

Layer (Type)	Output Shape	Parameters	Activation Function
Global Avg Pooling 2D	512	0	–
Dropout	512	0	–
Dense	100	51300	ReLU
Dense	1	100	Sigmoid

#### 3.5.1. Experimental setup.

The model was trained end-to-end using the Adam optimizer [43] for 20 epochs. Binary cross entropy loss was calculated. The model which had the lowest validation loss was selected [44]. The number of epochs, learning rate, and batch size were experimentally set to 20, 1E-3, and 32, respectively [45,46]. Table 5 shows values of other parameters of the Adam optimizer. Moreover, the proposed model training benefits from Intel(R) Core(TM) i9-13905 @ 2.6GHz with 32GB of RAM and a GPU of 8GB NVIDIA GeForec RTX 4060. For code implementation, we utilized the Windows 10 operating system and Python 3.10.6 as the programming language. Additionally, several Python libraries including Keras library [47] using Tensorflow 1.14 backend [48]. The model was trained on cloud Google Colaboratory [49] were used in the model training process.

**Table 5:** Adam optimizer parameters value.

Parameters	Values
Weight Decay Rate	0.0
Learning Rate	1E-3
Beta 1	0.9
Epsilon	1E-5
Beta 2 Momentum	0.999 0.9

## 4. Results

This section discusses the experimental setup and results. The results are presented in various forms to analyze. The results of the proposed model are compared with those of some other researchers. The advantages of the proposed research are also summarized.

### 4.1. Performance metrics

Four performance metrics were used for the analysis of the models; specificity, sensitivity, precision, and Area Under the Curve (AUC) [50–53]. The formulae used to calculate these criteria are shown in Equations 1–3, respectively.


$$Specificity=\frac{TN}{TN+FP}$$ (1)

$$Sensitivity=\frac{TP}{TP+FN}$$ (2)

$$\mathrm{Pr}ecision=\frac{TP}{TP+FP}$$ (3)

where TN, TP, FN, and FP are the number of True Negative cases, True Positive cases, False Negative cases, and False Positive cases, respectively [54–57]. TN is the number of negative (healthy) images that are truly labeled as healthy by the model, TP is the number of positive (disease) images that are truly labeled as a disease by the model, FN is the number of disease images that are falsely labeled as negative (healthy) by the model, whereas FP is the number of healthy images which are falsely labeled as positive (disease) by the model. AUC is the area under the Receiver Operating Characteristic (ROC) curve that is plotted between sensitivity and (1-specificity) [58,59]. This graph displays the model’s performance at different thresholds [60].

### 4.2. Predictive results of the proposed model against each disease

**Fig 5:**
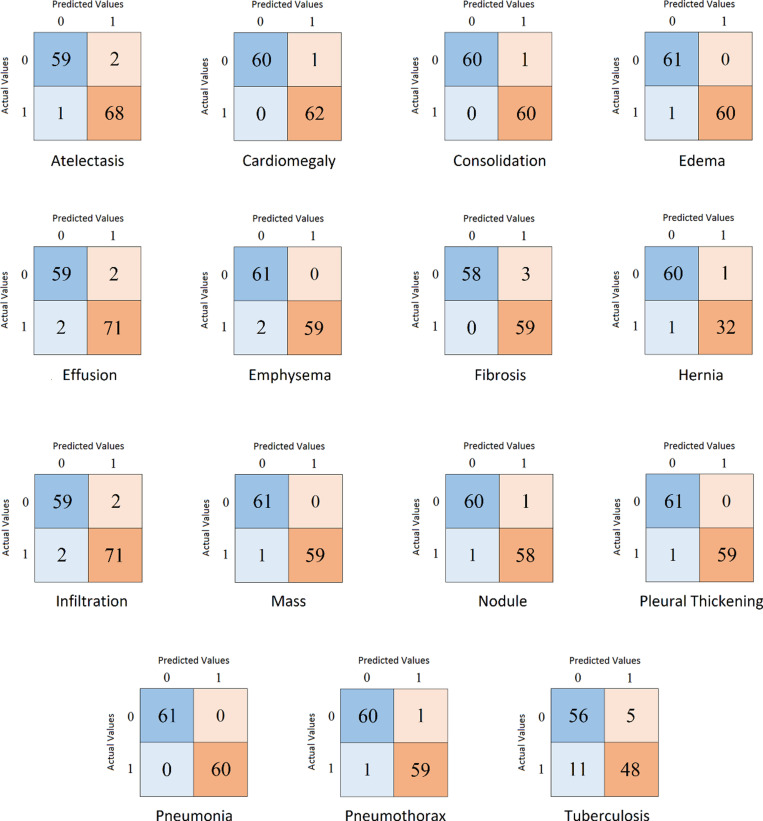
Confusion matrices of the diseases.

The specificities, sensitivities, precisions, and AUCs of the proposed model for different diseases are given in Table 6. It can be seen that the AUC for all the diseases except Tuberculosis is above 97%, with 100% being the highest and the overall AUC being 97.61%. As most of these diseases are very dangerous or fatal if not diagnosed at an early stage, the main focus of this research was to decrease the rate of False Negatives. The proposed model was successful in achieving this. The sensitivity of four of the diseases is 100%, and the others also have a sensitivity of 96% or higher, except for one disease. A low rate of False Negatives can also be observed in Fig 5, which shows confusion matrices of the diseases.

**Table 6:** Performance metrics for proposed model in percentage (%).

Diseases	Specificity	Sensitivity	Precision	AUC
Atelectasis	96.72	98.55	97.14	97.6
Cardiomegaly	98.36	100	98.41	99.2
Consolidation	98.36	100	98.36	99.2
Edema	100	98.36	100	99.2
Effusion	96.72	97.26	97.26	97
Emphysema	100	96.72	100	98.4
Fibrosis	95.08	100	95.16	97.5
Hernia	98.36	96.67	98.31	97.52
Infiltration	96.72	97.26	97.26	97
Mass	100	98.33	100	99.2
Nodule	98.36	98.31	98.31	98.3
Pleural Thickening	100	98.33	100	99.2
Pneumonia	100	100	100	100
Pneumothorax	98.36	98.33	98.33	98.3
Tuberculosis	91.80	81.36	90.57	86.6
**Overall**	**97.92**	**97.30**	**97.94**	**97.61**

**Fig 6:**
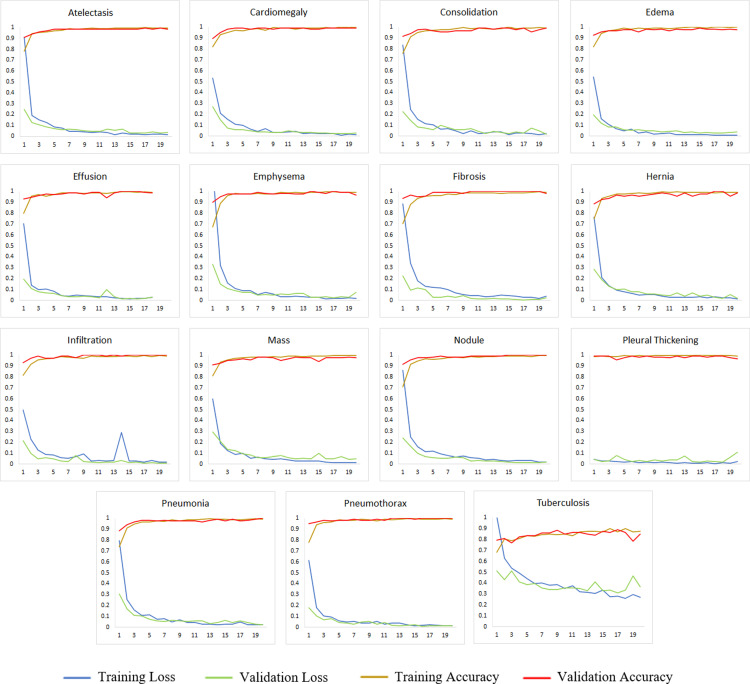
Graphs of training and validation accuracy and loss of the proposed model.

Fig 6 shows graphs of training and validation accuracy and loss of the proposed model. To avoid over-fitting, the model was trained up to the 20th epoch with a batch size equal to 32. It can be observed that the proposed model shows a fast-training process. Consequently, it also caused a rapid increase in the validation accuracy per epoch. The same is the case with the training and validation loss. A rapid decrease can be seen per epoch before it reaches a very low value. Moreover, on average, the proposed model took 540.003 seconds to extract features and then train for each disease. This training time helped us to achieve one of our objectives which was for the model to have very little execution time.

## 4.3. Discussion

The performance of the proposed model using VGG16 was compared to pre-trained DenseNet121 and VGG19. The fully connected layers of this model were replaced by the layers used in the classification stage of the proposed methodology. The weights of the layers before the fully connected block were frozen. Moreover, the performance was also compared to the original VGG16 model. Table 7 shows a comparison of performance in terms of the AUC of the proposed model with DenseNet121, VGG19, and original VGG16. The higher AUC for each disease is in bold. It can be observed that the AUC of the proposed model is higher for all diseases except Atelectasis, Infiltration, and Nodule, as compared to other models.

**Table 7:** Comparison of AUC (%) of proposed model with DenseNet121.

Diseases	AUCs of the Models			
**DenseNet121**	**VGG19**	**VGG16 (Original)**	**VGG16 (Proposed)**
Atelectasis	96.4	94.2	**98.2**	97.6
Cardiomegaly	98.4	98.4	94.5	**99.2**
Consolidation	94.2	98.7	93.4	**99.2**
Edema	97.5	96.4	96.4	**99.2**
Effusion	**97**	**97**	95.5	**97**
Emphysema	**98.4**	94.5	94.5	**98.4**
Fibrosis	**97.5**	94.5	**97.5**	**97.5**
Hernia	91.9	**97.5**	93.2	**97.5**
Infiltration	**98.6**	91.5	96.5	97
Mass	90.8	90.8	93.1	**99.2**
Nodule	**99.2**	97.5	94.3	98.3
Pleural Thickening	98.3	95.5	97.7	**99.2**
Pneumonia	99.2	98.5	93.5	**100**
Pneumothorax	97.5	94.6	**98.3**	**98.3**
Tuberculosis	77.3	77.3	82.4	**86.6**

Table 8 shows a comparison of performance in terms of the AUC of the proposed model with some state-of-the-art models for the detection and classification of multiple pulmonary diseases. The highest AUC for each disease is in bold. It can be observed that the AUC of the proposed model is the highest for all diseases except Atelectasis, Hernia, and Tuberculosis, as compared to other state-of-the-art models. However, the results of the proposed model for Atelectasis, Hernia, and Tuberculosis are still promising and comparable. It also shows the number of diseases each model can detect and classify and the overall AUC. It can be observed that the proposed model detected and classified a greater number of diseases as compared to all of these state-of-the-art models, and it also had the highest overall AUC.

**Table 8:** Comparison of the proposed model with other state-of-the-art models.

Diseases	AUCs of the Models (%)								
[61]	[62]	[63]	[19]	[64]	[65]	[66]	[67]	**Proposed**
Atelectasis	82.63	85	87	80.94	92.6	99.16	79.4	83.6	**97.6**
Cardiomegaly	87.32	95	97	92.48	88.2	99.1	89.6	91.7	**99.2**
Consolidation	74.06	85	–	79.01	–	**–**	79.8	81.5	**99.2**
Edema	88.47	96	–	88.78	88.7	**–**	87.0	90.1	**99.2**
Effusion	89.39	91	89	86.38	88.4	**–**	88.2	88.9	**97**
Emphysema	95.95	94	94	93.71	89.7	**–**	91.8	94.8	**98.4**
Fibrosis	80.03	87	–	80.47	87.3	**–**	80.3	86.2	**97.5**
Hernia	82.95	**99**	90	91.64	87.2	–	87.6	94.7	97.52
Infiltration	73.25	75	–	73.45	84.4	**–**	70.5	71.7	**97**
Mass	84.79	91	–	86.76	86.6	**–**	84.4	86.3	**99.2**
Nodule	76.33	81	69	78.02	85.3	**–**	75.2	82.4	**98.3**
Pleural Thickening	80.92	84	–	80.62	88.6	98.42	77.9	80.6	**99.2**
Pneumonia	77.84	81	–	76.80	87.3	**–**	76.3	78.3	**100**
Pneumothorax	90.84	93	–	88.87	89.6	**–**	87.8	89.3	**98.3**
Tuberculosis	–	–	94	–	–	**100**	–	–	86.6
Overall AUC	83.20	88.36	88.57	84.14	88.00		82.6	85.7	**97.61**
No. of Diseases	14	14	7	14	13	4	14	14	**15**

The proposed model has proven to be very efficient in detecting and classifying pulmonary diseases using chest radiographs. The advantages of this paper include that chest radiographs have been used as it is a cheaper, easier, faster, and less harmful method than CT scans. The proposed method provides an end-to-end structural model that does not use manual selection and feature extraction methods. It can detect and classify fifteen different pulmonary diseases. The model works efficiently with very little execution time (approx. 540.003 seconds). Moreover, the performance results are quite high.

## 5. Conclusion

Pulmonary diseases are among the leading causes of disabilities and deaths worldwide. It can be avoided by early detection and proper treatment of the disease. A deep transfer learning-based model was proposed in this paper, which detects fifteen different pulmonary diseases, including Atelectasis, Cardiomegaly, Consolidation, Edema, Effusion, Emphysema, Fibrosis, Hernia, Infiltration, Mass, Nodule, Pleural Thickening, Pneumonia, Pneumothorax, and Tuberculosis using chest radiographs. The results of the proposed model are very promising. The overall specificity of 97.92%, the sensitivity of 97.30%, the precision of 97.94%, and the AUC of 97.61% have been achieved. This model can help doctors in making decisions in clinical practice. However, it must be integrated as a decision-support tool rather than a standalone replacement for radiologists, while adhering to ethical standards and regulatory requirements.

There are a few limitations in the study. The first limitation is that no advanced preprocessing technique has been used. Future work will focus on improving the performance of this model by using some preprocessing techniques, such as cropping lungs from radiographs or extracting Regions of Interest (ROIs) from radiographs. This model can also be tried for the detection and classification of other diseases or from other modalities. Another limitation is that only AP and PA view chest X-rays were used in this paper. Lateral view chest X-rays were not included. Future work will also include them as they can be very helpful in diagnosing various diseases. Additionally, including patient history along with X-rays can also aid in improved diagnosis.

Public datasets often have limitations, including demographic biases, scanner variability, and insufficient annotations, which may affect the generalizability of models in diverse clinical settings. Future work will apply cross-dataset validation to overcome this issue. Moreover, the reliance on weak or automated labels derived from radiology reports, rather than direct expert annotations, introduces noise into the data and may diminish the accuracy of the resulting models. To enhance the reliability, manual annotation by medical experts will be done in future work.
